# Selective Inhibition of CK2: Emerging Strategies and Future Directions

**DOI:** 10.1021/acs.jmedchem.6c00643

**Published:** 2026-07-28

**Authors:** Aryaman R. Sokhal, Sona Krajcovicova, Jessica Iegre, Paul Brear, Claudia De Fusco, Paul A. Glossop, Darren Cawkill, Marko Hyvönen, David R. Spring

**Affiliations:** † Yusuf Hamied Department of Chemistry, 2152 University of Cambridge, Cambridge CB2 1EW, United Kingdom; ‡ Department of Organic Chemistry, Faculty of Science, Palacky University, Olomouc 77900, Czech Republic; § Department of Biochemistry, University of Cambridge, Cambridge CB2 1GA, United Kingdom; ∥ Sandexis Medicinal Chemistry Ltd, Discovery Park, Sandwich CT13 9FF, United Kingdom; ⊥ Apollo Therapeutics Ltd, Cambridge CB1 2JH, United Kingdom

## Abstract

CK2 is a constitutively active serine/threonine kinase implicated in cancer, viral infection, and neurodegeneration, but its conserved ATP-binding site and complex holoenzyme assembly have long made selective inhibition challenging. This Perspective discusses three complementary strategies for selective CK2 inhibition: targeting the CK2α/β interface with small molecules, disrupting the same interface with conformationally constrained peptides, and exploiting the cryptic αD pocket for dual-site inhibitor design. CK2α/β-interface inhibitors, including CAM187, CAM7117 and P8C9, established structurally validated routes to modulate holoenzyme assembly and β-dependent substrate phosphorylation. Validation of the αD pocket as a ligandable cryptosteric site enabled dual-site ligands such as CAM4066 and αD-directed inhibitors such as CAM4712, followed by related advances including AB668, KDX1381 and Biv5. These efforts culminated in APL-5125, a highly selective, subnanomolar dual-site inhibitor now in Phase 1/2 clinical evaluation.

## Graphical abstract

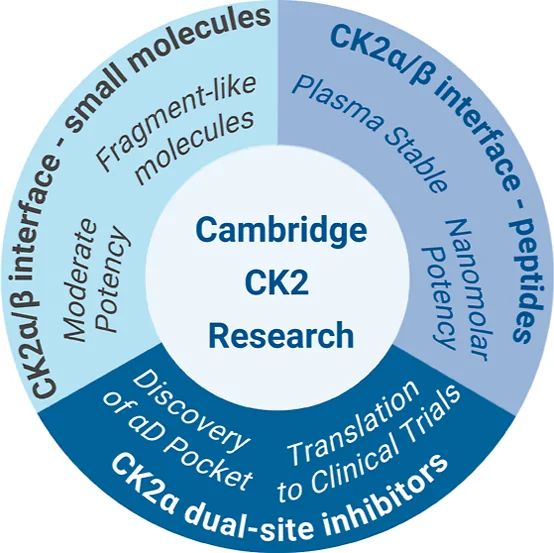

## 1 Introduction

Cancer is a complex group of diseases defined by uncontrolled proliferation, evasion of apoptosis, metabolic reprogramming, and metastasis. These hallmarks are driven by the dysregulation of signaling pathways often controlled by protein kinases.^1^ These kinases orchestrate vital cellular processes, and their aberrant activity is frequently implicated in oncogenesis and tumor progression. One such example is casein kinase 2 (CK2), a serine/threonine protein kinase formerly classified within the CMGC family.^2,3^ Evolutionarily conserved, CK2 is a central regulator across diverse cellular contexts, influencing processes such as growth, division, and apoptosis.^4,5^ Unlike most kinases, CK2 is constitutively active, functioning without upstream signals,^6^ a trait that facilitates its pathological role in cancer by amplifying oncogenic signaling.^7^ CK2 promotes tumorigenesis by phosphorylating key proteins like Akt,^8^ β-catenin,^9^ and Janus kinase 2,^10^ while simultaneously inhibiting tumor suppressors such as phosphatase and tensin homologue.^11^ Consequently, elevated CK2 expression creates an environment favorable to malignant transformation and is a documented marker in glioblastoma, cholangiocarcinoma, and breast cancers.^12^ Beyond oncology, CK2 is implicated in neurodegenerative disorders like Alzheimer’s, Parkinson’s, and amyotrophic lateral sclerosis,^12,13^ as well as viral pathogenesis, notably interacting with the SARS-CoV-2 nucleocapsid protein.^14^ Consequently, it is a key therapeutic target that has been explored widely for the development of inhibitors.

CK2 is a heterotetrameric enzyme complex (Figure 1A) consisting of two highly similar α or α′ catalytic kinase subunits and two regulatory β subunits. The α subunits mediate phosphorylation of target proteins, while the β subunits govern stability and substrate specificity.^15^ Therefore, selective modulation of the CK2α/β interaction represents a therapeutically relevant strategy for controlling β-dependent substrate phosphorylation and CK2 localization without directly targeting the highly conserved ATP-binding site. In many cancers, an imbalance, specifically overexpression of CK2α alongside reduced CK2β levels, further drives oncogenic signaling.^16^

**Figure 1:**
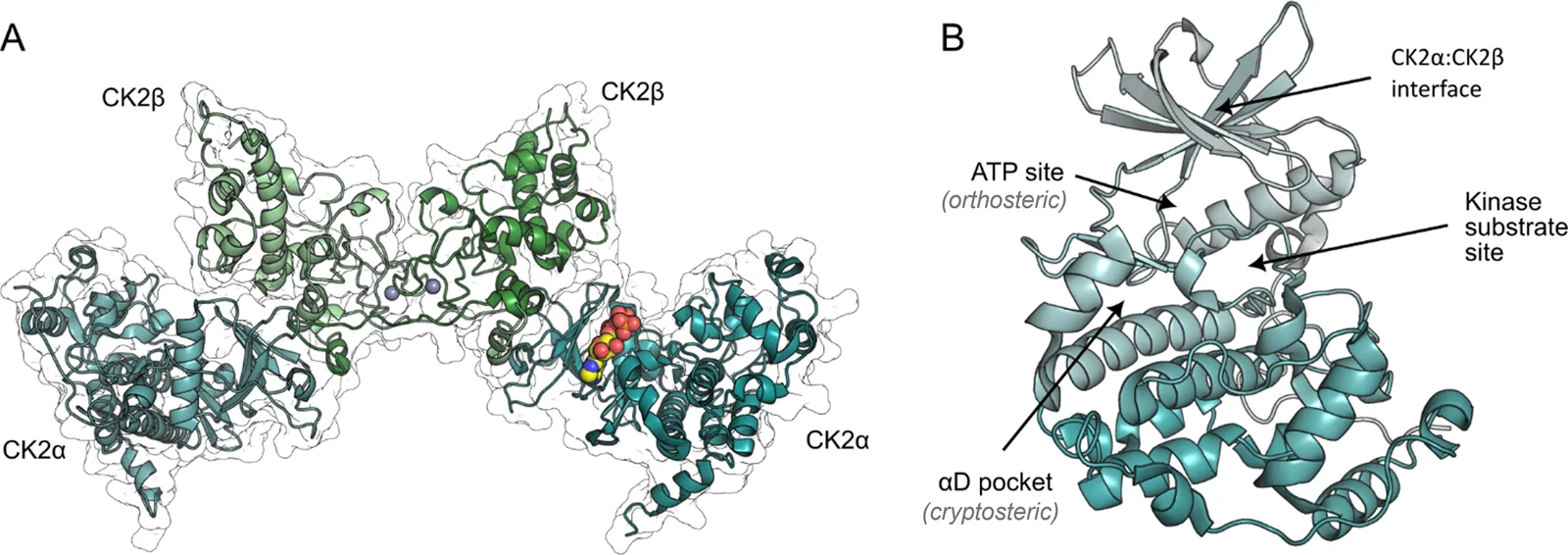
(A) The CK2 holoenzyme structure, showing two catalytic CK2α subunits (darker and light blue) and two regulatory CK2β subunits (darker and lighter green), along with surface outline for the complex (PDB ID: 1JWH). (B) Kinase subunit CK2α, with the key binding sites indicated by arrows. The cartoon is colored from light blue at the N-terminus to darker blue at the C-terminus (PDB: 5OS7).

The ATP-binding site of the CK2α subunit houses the adenosine ring within a hydrophobic pocket formed by Ile174 and Val66. The kinase activity is facilitated by Lys68, a conserved catalytic residue, hydrogen bonding between the phosphate group and the backbone of His160, and a water-mediated interaction with Ser51.^17^ Early pharmacological efforts focused on competitive inhibition of this site.^18^ However, often suffered from poor selectivity due to the highly conserved nature of the ATP site in protein kinases.

In 2012, the Hyvönen and Spring groups, supported by the group of Chris Abell, began a fragment-based program aimed at addressing the long-standing challenge of selective CK2 inhibition.^19,20^ This work initially explored the CK2α/β interface as a noncatalytic site for modulating CK2 function, before expanding toward constrained peptide inhibitors and, subsequently, dual-site ligands that exploit the cryptic αD pocket, utilizing a novel dual orthosteric-cryptosteric binding approach. Rather than providing a comprehensive survey of all CK2 inhibitors,^18,21^ this Perspective highlights these specific design advances and their relationship to subsequent developments in the field. Together, they illustrate distinct routes to selectivity beyond conventional ATP-competitive inhibition: (A) disruption of the CK2α/β interface with small molecules, (B) development of constrained peptide mimics of the CK2β interface motif, and (C) exploitation of the cryptic αD pocket for dual orthosteric–cryptosteric inhibition. Together, these examples show how fragment-based discovery, structural biology, and peptide-constraining chemistry have been used to overcome the limitations of highly conserved kinase ATP sites and to generate increasingly selective CK2 inhibitors.

## 2 Strategy A: Disrupting the CK2 α/β Interface with Small Molecules

Early efforts to achieve CK2 selectivity targeted the α/β subunit interface with small molecules to modulate β-dependent signaling, which governs substrate specificity and localization.^22^ Because the CK2α/β interface lies outside the conserved ATP-binding site, it offers a mechanistically distinct route to CK2 modulation compared with conventional Type I ATP-competitive inhibitors, with the potential to improve selectivity by targeting holoenzyme assembly, β-dependent substrate phosphorylation, and CK2 localization. Raaf et al. first provided proof of concept with compound **1**, which bound both the ATP site and at a CK2α/β interface pocket.^23^ Subsequent identification of tetrahydrocarbazole derivative W16 (**2**; IC_50_ = 30–40 μM)^24^ by Laudet et al. and Eriochrome Black A (**3)** and Eriochrome Black T (**4**; IC_50_ = 0.4 μM) by Moucadel et al.^25^ further suggested this region was a tractable site (Figure 2A). Despite this promise, these inhibitors faced significant hurdles: crystallographic data were not available to confirm the exact binding modes for compounds **2**-**4**, and their large, polar scaffolds resulted in poor physicochemical properties, limiting their use as therapeutic leads or chemical probes.

**Figure 2:**
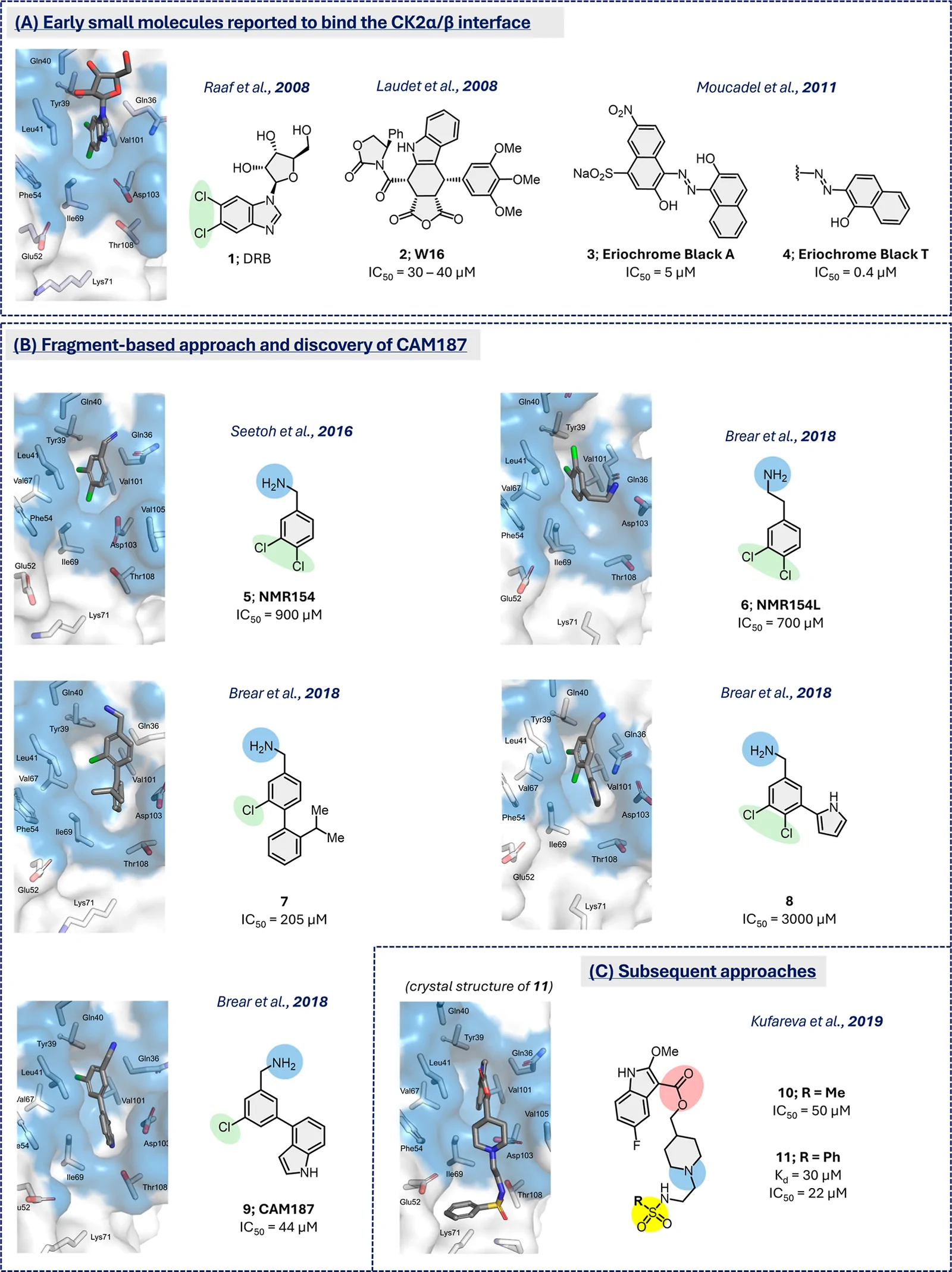
Small molecule CK2α/CK2β inhibitors. (A) Early small molecules reported or proposed to bind the CK2α/β interface. DRB (**1)** was the first compound that was shown to bind to CK2α/β interface (PDB: 3H30). **2**-**4** bind to CK2α with low-to-medium micromolar affinity but their complexes with CK2α have not been characterized structurally. (B) Fragment-based approach leading to CAM187 (**9**). Initial fragments **5** (PDB: 5NQC) and **6** (PDB: 5CLP) bound to a site analogous to **1**, but in different orientations. Bicyclic compounds **7** (PDB: 5OS7), **8**, and **9** (PDB: 6GIH) occupy a longer hydrophobic groove at the interface. (C) Subsequent efforts to inhibit the CK2α/β interface with small molecules. Compound **11**, bearing a benzene-substituted sulfonamide, binds to a more elongated interface, with terminal phenyl group inserting itself into a new pocket (PDB: 6FVG). In all structures the side chains of the residues interacting with CK2β in CK2 holoenzyme are shown as sticks (with nitrogen in blue, oxygen in red, chlorine in pale green and sulfur in yellow). The translucent surface on CK2α is colored blue where the CK2β peptide would be closest to the protein, based on superimposed complex with *Pc* peptide (PDB: 4IB5).

Building on these early insights, our groups pursued two complementary strategies to develop more potent, structurally validated CK2 inhibitors. The first strategy targeted the α/β subunit interface to disrupt holoenzyme assembly, affecting both catalytic function and localization. While this work validated the interface as an allosteric site, it also highlighted the need for ligands with improved potency and well-defined binding modes. To overcome previous scaffold limitations, we adopted a fragment-based approach targeting low-molecular-weight molecules compliant with the *rule of three*.^26^ Similar to Lipinski’s *rule of five* ^27^ this strategy ensures favorable physicochemical profiles and allows for efficient sampling of chemical space during structure-guided optimization. According to this rule, suitable fragments typically possess a molecular weight below 300, a clogP ≤3, and no more than three hydrogen-bond donors or acceptors. Such fragments can serve as starting points for the development of more drug-like inhibitors targeting the α/β interface.^28^ An initial screen identified NMR154 (**5**), which bound both the ATP site and the α/β interface with an IC_50_ of 900 μM (Figure 2B).^29^ A key interaction was an electrostatic contact between the amine of **5** and Asp37 of CK2α, the same residue that forms a salt bridge with Arg186 of CK2β. This observation provided the basis for subsequent optimization.^28^ The first analogue, NMR154L (**6**), replaced the methylamino moiety of compound **5** with an ethylamino group. While it retained the Asp37 interaction, it offered only a modest potency improvement (IC_50_ = 700 μM).

Consequently, the methylamino group was retained for subsequent designs (Figure 2B), as amine substitutions failed to enhance binding. Focus then shifted to aromatic core modifications to improve engagement of the α/β interface. *Para*-substitution relative to the methylamino group produced a series of biphenyl analogues with improved potencies (<500 μM), notably compound **7** with an IC_50_ of 205 μM, which was also characterized using crystallography (Figure 2B). However, these analogues also engaged the αD pocket, a narrow, hydrophobic site distinct from the α/β interface (and discussed later in detail), raising concerns about their selectivity for the α/β interface. This observation prompted the hypothesis that *meta*-substitution, rather than *para*-, might improve α/β interface selectivity. To test this, compound **8** bearing a *meta*-linked pyrrole substituent was synthesized (Figure 2B). Although it displayed lower potency (IC_50_ = 3 mM), it no longer occupied the αD pocket, indicating improved selectivity for the α/β interface. Building on this finding, we proposed introducing a bulkier indole group at the meta-position to better exploit the hydrophobic environment, while removing the *para*-chloro substituent to reduce lipophilicity. This strategy led to CAM187 (**9**), which achieved a binding IC_50_ of 44 μM and, importantly, was confirmed by crystallography to bind selectively at the α/β interface (Figure 2B).

While the potency of **9** was comparable to **2** and lower than **3** or **4**, it marked a significant advance due to its selective binding, favorable physicochemical profile and crystallographic validation. Compound **9** established the first clear structural foundation for the rational design of selective small-molecule inhibitors targeting the CK2 α/β interface. Since its publication, other groups have further progressed the development of potent binders for this site. The first of these advancements came from Kufareva et al.,^30^ who underscored the challenges associated with this target site, particularly its shallow topology and pronounced conformational flexibility. To address this, the authors used a “fumigated” model of CK2α to expose cryptic pockets for virtual screening. Candidates were filtered based on physicochemical properties and their ability to reach Val112 at the pocket base. From this, compound **10** was identified, inhibiting phosphorylation of its substrate Olig-2 with an IC_50_ of 50 μM by disrupting holoenzyme assembly (Figure 2C). Selectivity was quantified using the Gini coefficient, where 0 represents no selectivity and 1 represents full selectivity;^31^ compound **10** proved highly selective (0.81),^19,20^ described more in the next section. SAR studies led to CCH507 (**11**), which features a phenyl substituent on the sulfonamide (Figure 2C). This modification improved binding affinity at the interface (K_d_ = 30 μM) and inhibited phosphorylation of CK2β-dependent substrates with an IC_50_ of 22 μM. Crystallography studies showed the phenyl ring occupying an additional pocket at the end of the CK2β binding groove. Binding of **11** induced a conformational change in the β4-β5 loop, expanding the pocket. In cellular assays, **11** disrupted holoenzyme formation, confirmed by coimmunoprecipitation and proximity ligation, leading to reduced substrate phosphorylation and cell viability. This provides strong biological validation for the α/β interface as a therapeutic target. Despite this, no further small-molecule inhibitors for this interface have been reported, highlighting the ongoing difficulty of targeting protein–protein interactions.

## 3 Strategy B: Disrupting the CK2 α/β Interface with Constrained Peptides

While fragment-based and virtual screening have yielded valuable small-molecule binders for the CK2 α/β interface, their potencies and drug-like properties require further optimization. Consequently, peptide-based inhibitors have emerged as a complementary strategy, offering higher affinity by mimicking the natural interface. Crystal structures show that the α/β interaction is mediated by a linear epitope on CK2β.^32^ Mutagenesis identified Tyr188 and Phe190 as essential hydrophobic “hotspots”; substituting these residues disrupts subunit association and reduces the phosphorylation of β-dependent substrates (Figure 3A). Laudet et al.^33^ provided proof of concept for disrupting the CK2 α/β interfaceusing a cyclic peptide ***Pc*** (**12**), constrained by a disulfide bridge between terminal cysteines (Figure 3A). **12** contained eight residues from CK2β (residues 186-193, RLYGFKIH) flanked by cysteines and glycines to facilitate cyclization. **12** inhibited CK2α/β association with an IC_50_ of 3 μM (an order of magnitude more potent than its linear analogue) and reduced phosphorylation of the β-dependent substrate Olig-2 in a biochemical assay.

**Figure 3:**
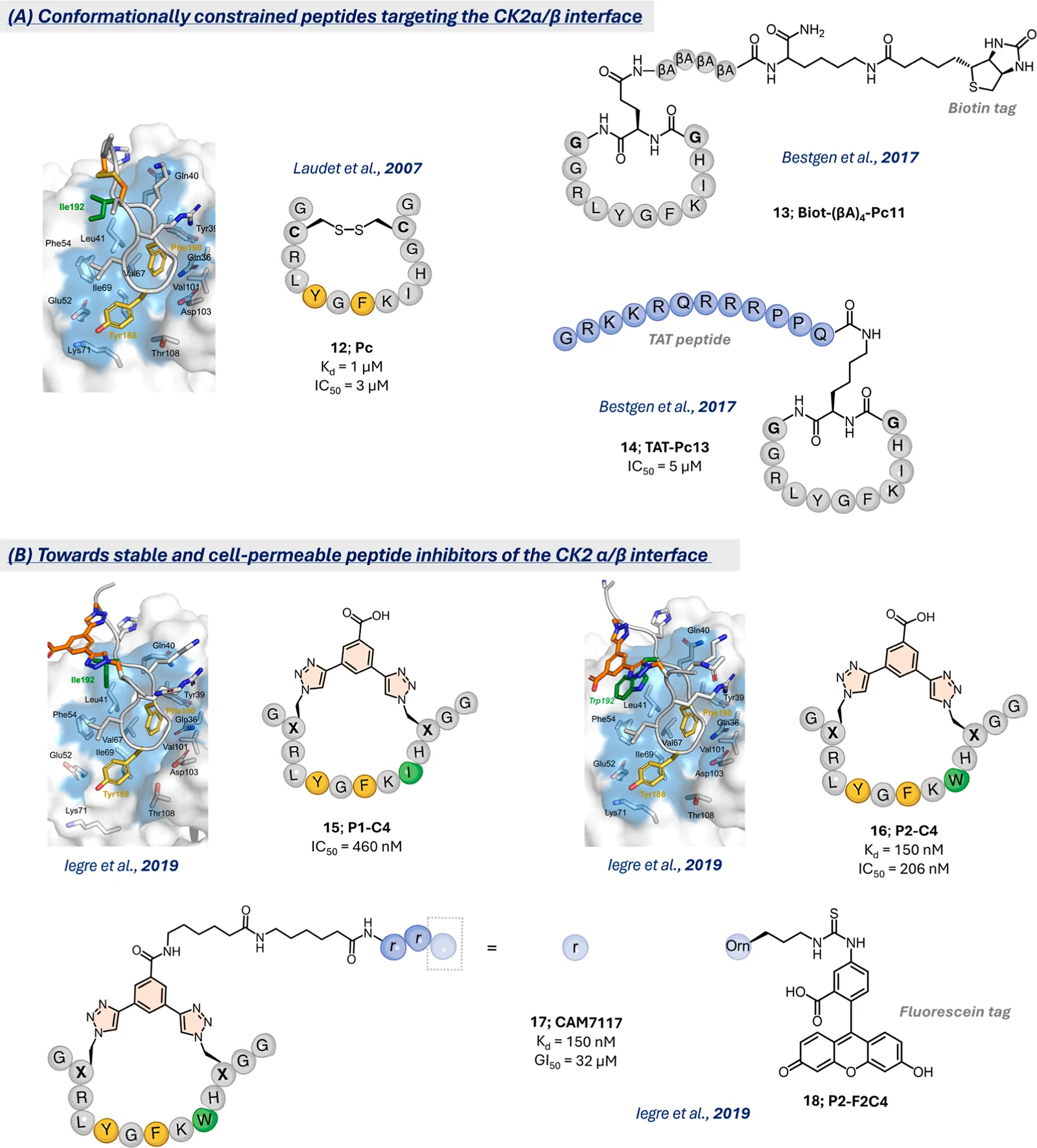
Macrocyclic CK2α/CK2β inhibitors*.* **(A)** Head-to-tail cyclized, conformationally constrained peptides targeting the CK2α/β interface. Peptide ***Pc*** (**12**) and its binding mode (PDB:4IB5) show a disulfide-constrained scaffold with key Phe and Tyr residues (yellow). Biot-(βAla)_4_-Pc11 (**13**) demonstrates that head-to-tail cyclization can substitute for disulfide stapling, with a biotin tag for pull-down assays. TAT-Pc13 (**14**) incorporates an N-terminal TAT sequence (blue) to enhance uptake of the 13-mer. **(B)** Development of CAM7117 (**17**), a stable and cell-permeable CK2α/β interface inhibitor. Stapled peptide P1–C4 (**15**), derived from **12** using a two-component CuAAC benzoic-acid staple, binds the interface (PDB:6Q38). Optimized **16** replaces Ile9 with Trp, enabling π–π stacking with the staple and improving affinity (PDB:6Q4Q). **17** adds a spacer and a (d)-arginine tripeptide to promote cellular uptake. Fluorescent analogue **18** enables imaging of localization and uptake. Key interacting residues are colored yellow (Tyr and Phe) and green (Ile or Trp), while the cross-links of different types are colored orange. Abbreviations: βA = beta-alanine, r = d-Arg, Orn = ornithine, TAT = trans-activating transcriptional activator, X = 3-azido-l-alanine. Blue surface coloring defined as in Figure 2.

Shorter disulfide-linked ***Pc*** variants, down to CK2β sequence RLYGFKI, retained comparable activity, whereas the peptide where the terminal isoleucine was deleted was inactive.^34^ To improve stability under reductive conditions, a head-to-tail cyclized analogue, Biot-(β-Ala)_4_-Pc_11_ (**13**), was prepared and shown to bind CK2α in pull-down assays both *in vitro* and in CK2β-deficient cell extracts. Further optimization produced the cell-permeable TAT-fused analogue TAT-Pc_13_ (**14**), which inhibited phosphorylation of eIF2β with an IC_50_ of 5 μM and induced cell death across multiple cancer cell lines, highlighting its potential as a therapeutic candidate (Figure 3A). Although these peptides demonstrated promising activity, no structural validation or stability data were reported.

To address these limitations, our groups sought to develop a potent and stable conformationally constrained peptide using their two-component copper-catalyzed azide–alkyne cycloaddition (CuAAC) peptide-stapling methodology.^35^ This strategy had already been validated in other systems as a means of conferring stability onto cyclic peptides.^36–38^ Accordingly, the group applied this approach to CK2α/β interface-targeting peptides.^39^

**12** was selected as the starting point, and molecular modeling indicated that amino acids at positions 2 and 11 would be optimal for stapling as they were not part of the native CK2β. Thus, Cys2 and Gly11 of peptide **12** were replaced with azido amino acids bearing one-carbon side chains. After screening different aliphatic and aromatic linkers, P1–C4 (**15**) emerged as the most effective, showing a potency of 460 nM, twice as strong as **12** (Figure 3B). The crystal structure of **15** bound to the interface revealed that Ile9 could be substituted with an aromatic residue to enable π–π stacking with the benzene ring of the linker. This additional interaction was expected to reduce peptide flexibility and lower the entropic penalty of binding. Replacing Ile9 of the peptide (equivalent to Ile192 in CK2β) with a tryptophan yielded P2–C4 (**16**), which indeed demonstrated increased affinity (K_d_ = 150 nM; Figure 3B). In vitro assays confirmed that both **15** and **16** inhibited holoenzyme assembly, and **16** further reduced phosphorylation of the CK2β-dependent substrate eIF2β with an IC_50_ of 206 ± 29 nM, outperforming all previously reported small molecules and peptides targeting the CK2α/β interface.

The next challenge was to achieve cell permeability. To this end, we employed a (d)-arginine tripeptide, previously shown in its (l)-form to enhance cell uptake of peptides.^37,40^ The use of the d-enantiomer provided additional proteolytic stability. The benzoic acid moiety was linked to the tripeptide via a spacer, then incorporated into the stapled peptide to generate CAM7117 (**17**; Figure 3B). Plasma stability assays showed that **17** was relatively stable, with 47% of the peptide intact after 24 h of incubation. In functional assays, **17** inhibited human osteosarcoma cell growth with a GI_50_ of 32 ± 2 μM, though activity was reduced in human colorectal cancer cells (Figure 3B).

A fluorescent analogue, P2–F2C4 **18**, bearing the fluorescein tag, was also synthesized for cellular studies. To investigate the discrepancy between in vitro potency and cellular activity, imaging experiments were performed with **18**. A substantial proportion of the peptide was found sequestered in the endoplasmic reticulum and Golgi apparatus. Moreover, because CK2α is constitutively active in its apo form, phosphorylation of β-independent substrates likely persisted, thereby limiting its cellular effect. Despite these challenges, the study demonstrated a clear strategy for generating stable, cell-permeable, multifunctional, and conformationally constrained peptides. **17** in particular showed strong promise as a chemical probe for disrupting the CK2α/β interface, marking an important advance over previous efforts. Encouraged by these results, we then aimed to develop a more efficient constrained peptide that retained the stability and cell permeability of **17** while being easier to synthesize.^41^ This was an important goal, as the synthesis of **17** required the use of non-natural D amino acids and a noncommercial dialkyne benzoic acid staple, which made the process low-yielding and impractical.

To improve binding, we hypothesized that reducing peptide size would limit conformational flexibility and enforce a more rigid, binding-competent geometry. Structural analysis of **17** indicated that only the central residues RLYGFKWH were essential for interaction at the CK2α/β interface. Modeling suggested optimal stapling between the residue preceding this motif and the *C*-terminal Trp, shortening the staple distance from 11.6 to 7.5 Å. Systematic truncation confirmed that while Arg1 and Trp7 were indispensable, His8 contributed little and could be omitted, yielding the minimal sequence RLYGFKW. Among the resulting cyclic analogues, P8C9 (**19**) showed the strongest binding (K_d_ = 750 nM), with 5-fold higher activity than **17** (Figure 3B). **19**, cyclized via oxidative coupling between an *N*-terminal homocysteine and the *C*-terminal Trp, also exhibited full plasma stability for over 24 h, marking a substantial improvement over its predecessor. The compact structure of **19** provided opportunities for further functionalization. As both the *N*-terminus and the Lys side chain were solvent-exposed, these sites were explored for conjugation. Addition of a fatty acid and polyethylene glycol (PEG) chain at the *N*-terminus produced FA-P8C9 (**20**), which retained binding affinity with only a modest decrease (K_d_ = 1.3 μM). Similarly, PEGylation of the Lys side chain yielded P8C9­[PEG] (**21**) with a K_d_ of 1.6 μM (Figure 4). Cellular assays revealed that unmodified **19** did not affect HeLa cell viability. Analogue **20** reduced cell viability, but this was attributed to nonspecific toxicity from the fatty acid-PEG moiety rather than on-target inhibition. In contrast, N-terminal attachment of well-characterized cell-penetrating peptides (CPPs), namely TAT or a triarginine tag, successfully conferred cell permeability and restored activity, with both analogues (**22** and **23**) significantly reducing cell viability (Figure 4).

**Figure 4:**
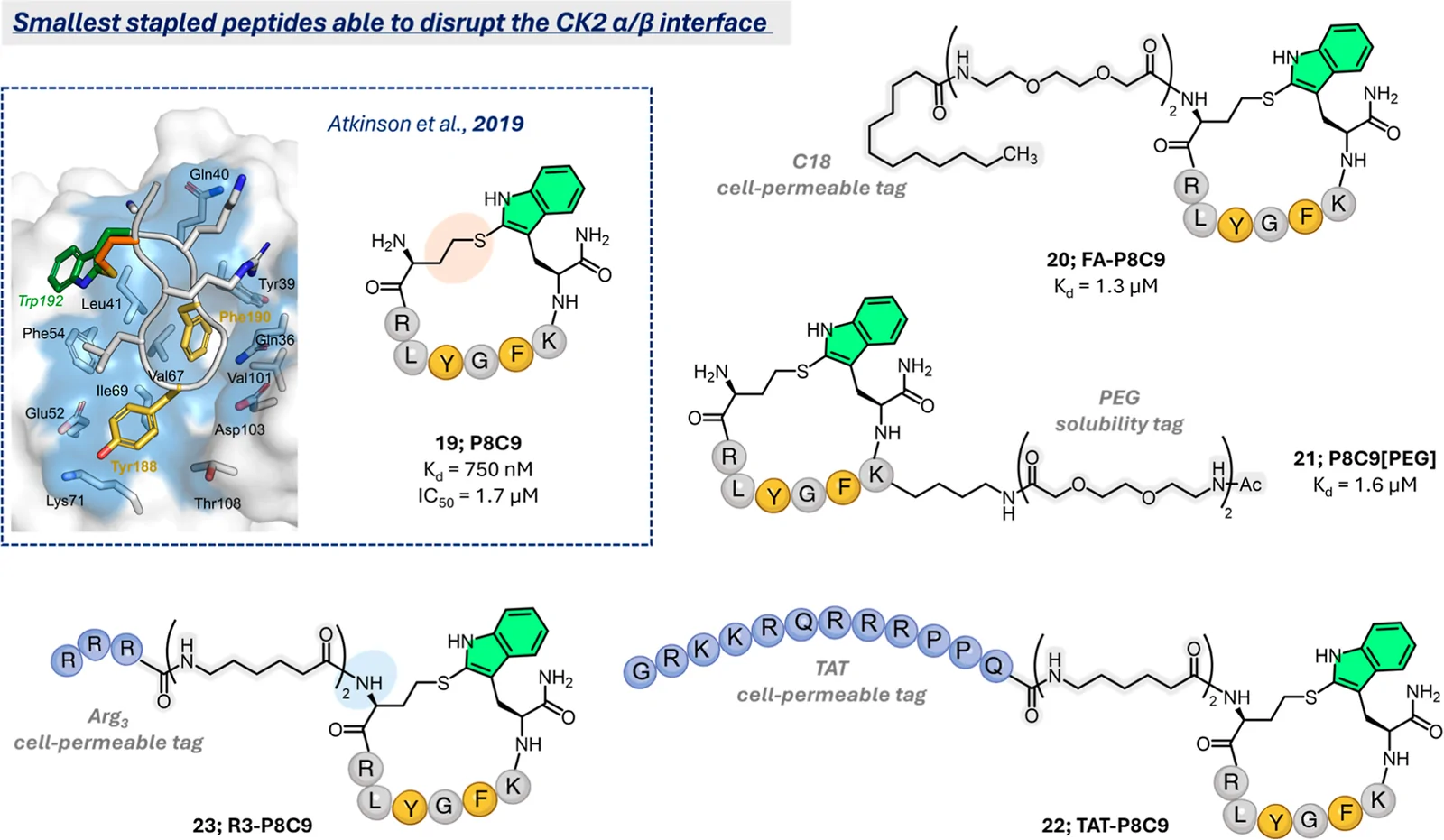
Small CK2α/CK2β inhibiting cyclic peptides. P8C9 (**19**) showing the homocysteine–tryptophan staple, and its functionalized analogues **20-23**. The crystal structure of **19** bound at the CK2α/β interface is also shown (PDB: 6YZH). Coloring the same as in Figure 3.

Overall, **19** and its CPP-functionalized derivatives **20-23** represent the smallest conformationally constrained peptides reported to disrupt the CK2α/β interface. They retain the binding mode of **17** while offering markedly improved plasma stability, simpler synthesis, and facile sites for further optimization. These features make **19** a promising new scaffold for the development of next-generation chemical probes and potential therapeutic leads.

## 4 Strategy C: Exploiting the Cryptosteric αD Pocket and Dual-Site Inhibition

While peptides and small molecules that target the CK2α/β interface have demonstrated the feasibility of disrupting holoenzyme formation, their relatively shallow binding pocket, limited potency, and cell permeability challenges have limited their progression. To overcome these shortcomings, an alternative strategy has been to design dual-site inhibitors that simultaneously engage the ATP-binding site of CK2α as well as an adjacent pocket. This approach combines the high affinity typically associated with ATP-competitive inhibitors with the selectivity gains afforded by targeting a second, less conserved site.

The first bivalent CK2 inhibitor was reported by Enkvist et al.,^42^ who exploited CK2’s preference for acidic substrates by linking an ATP-site ligand to a short acidic peptide. Using TBBi (**24**), a CK2 inhibitor (IC_50_ = 500 nM)^43^ but more potent against PIM1 (IC_50_ = 240 nM),^44^ they generated conjugates varying in sequence and linker length (Figure 5). The most active, ARC-1502 (**27**), containing a (Asp)_6_Lys chain, showed remarkable potency (K_d_ = 0.56 nM, IC_50_ = 2.7 nM) and selectivity (Gini = 0.616; 10/140 kinases inhibited >50% at 1 μM), validating the dual-site concept. Cozza et al.^45^ next developed K137-E4 (**26**) from K137 (**25**), a tetrabromobenzimidazole analogue (IC_50_ = 130 nM; Figure 5).^46^ Adding four glutamates improved potency 10-fold (IC_50_ = 25 nM) and conferred exceptional selectivity, though the charged peptide tail restricted activity to ecto-CK2, where inhibition impaired extracellular phosphorylation and cancer-cell migration. **27** and **29** were both characterized structurally, but while the halogenated ATP-site binding moieties are clearly seen in the structures, there was no density for the acidic substrate peptide-mimic.

**Figure 5:**
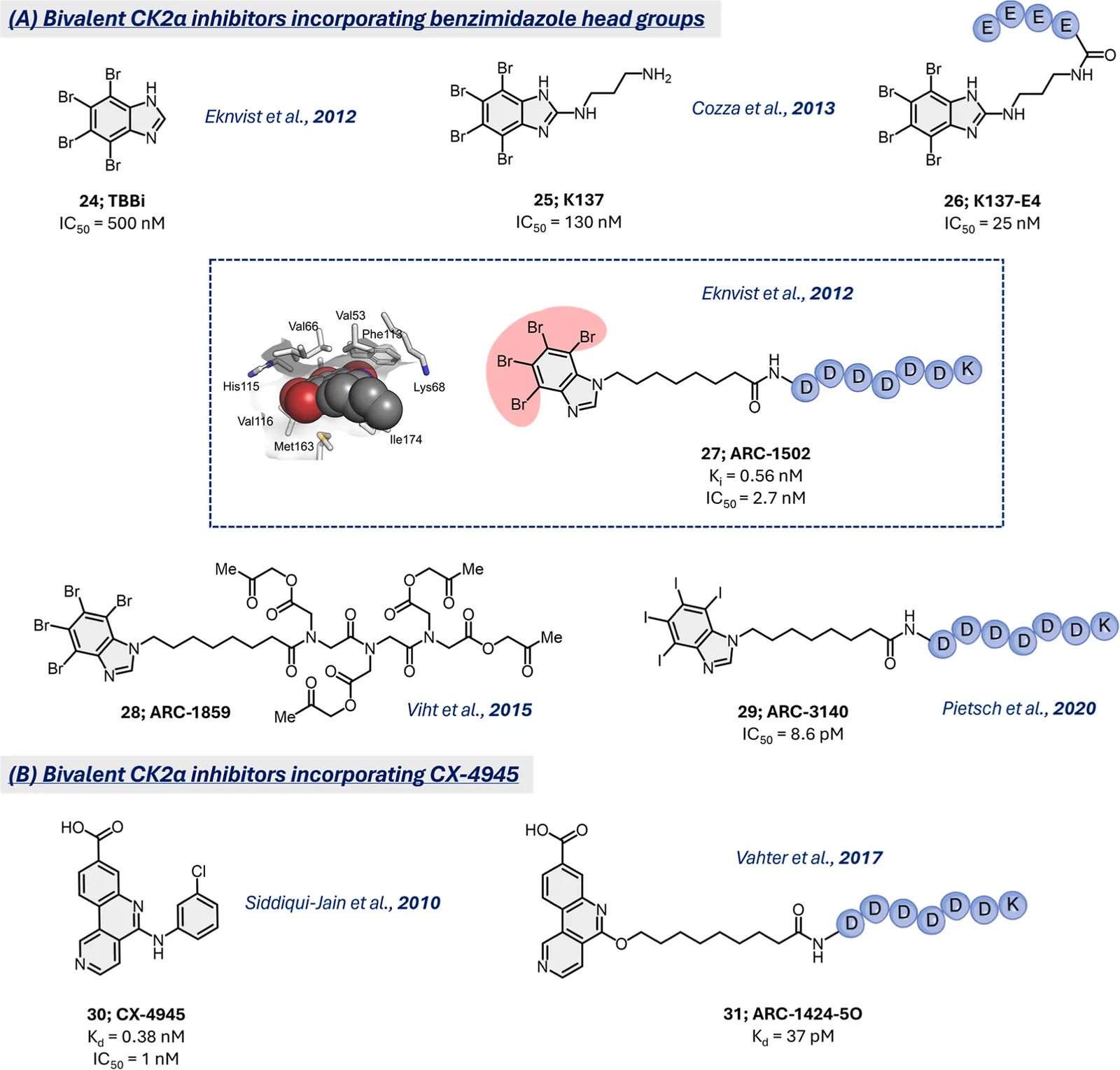
Bivalent CK2α inhibitors incorporating benzimidazole head groups or CX-4945 (**30**), tethered to acidic chains. (A) TBBi (**24**), an ATP-site ligand, and ARC-1502 (**27**), the first-generation bivalent ATP-binding site/substrate-binding site inhibitor (IC_50_ = 2.7 nM, K_i_ = 0.56 nM). The crystal structure of **27** bound to CK2α is shown (PDB: 6SPX). K137 (**25**) and its bivalent analogue K137-E4 (**26**), obtained by conjugation with a glutamate chain. ARC-1859 (**28**), a stable and cell-permeable acetoxymethyl ester prodrug of ARC-1502. ARC-3140 (**29**), a tetra-iodo analogue of ARC-1502, with markedly enhanced affinity. In addition to binding the ATP site, **29** also engages the CK2α/β interface (PDB: 6SPW). (B) CX-4945 (**30**), a CK2α ATP-binding site ligand that is used as the basis for ARC-1424**-**5O (**31**), a bivalent CK2α inhibitor that simultaneously engages the ATP-binding site and the substrate recognition pocket. (color coding: bromine as red, aspartic and glutamic acids as blue). In the structure of **27**, all the residues surrounding the ATP site of CK2α are shown as sticks and labeled.

To address poor permeability, **27** was converted into the acetoxymethyl-ester prodrug ARC-1859 (**28**).^47^ **28** retained potency, showed good plasma stability and uptake, and reduced CK2 substrate phosphorylation and HeLa cell viability, effectively resolving the permeability issue (Figure 5). Substituting bromine with iodine produced ARC-3140 ^48,49^ (**29**), enhancing affinity (K_d_ = 0.087 nM) and revealing an additional binding site at the CK2 α/β interface (K_i_ = 4.8 μM; Figure 5). Notably, **27** also inhibited this interaction in solution (K_i_ = 6.4 μM). **29** thus represents a rare multisite inhibitor simultaneously engaging the ATP site, substrate pocket, and interface. Finally, using Silmitasertib (CX-4945; **30**), a potent but nonselective CK2 inhibitor (IC_50_ = 1 nM) previously in clinical trials,^50–52^ as the ATP-site anchor, a poly aspartic acid chain was conjugated without disrupting its Lys68 salt bridge (Figure 5).^53^ The resulting ARC-1424**-**5O (**31**) achieved exceptional affinity (K_d_ = 37 pM), among the highest for CK2 inhibitors, though its selectivity remained comparable to **30** (Figure 5).

While the bivalent approach of targeting CK2α through both the ATP-binding site (orthosteric) and the substrate recognition site represented an important step toward selectivity, it was still limited by off-target activity against kinases with similar substrate-binding regions and poor cellular uptake. Thus, identifying a unique specificity-determining site in CK2 was seen as critical for further development. In 2016, our groups reported the experimental validation and exploitation of a cryptic pocket in CK2α, termed the αD pocket, located behind the αD helix (Asp120**-**Thr127).^19,20^ This cryptosteric site had previously been suggested in two crystallographic studies.^54,55^ However, these earlier studies did not reveal the full extent of the pocket or establish its ligandability. The pocket was subsequently identified as a tractable ligand-binding site during a serendipitous high-concentration crystallographic screen for compounds targeting the CK2α/β interface. The promiscuous fragment, 3,4-dichlorophenylethylamine (**6**; Figure 2B), was found to bind not only the α/β interface but also the ATP-binding site and in a previously unseen cryptic pocket just below the ATP site. This pocket was revealed only when the αD-helix moved from its usual position, with the fragment displacing Tyr125. To exploit the deep, hydrophobic nature of this αD pocket, fragment optimization led to the development of a biphenyl derivative **32** (Figure 6), which bound the pocket with a K_d_ of 270 μM. We hypothesized that linking such an αD binder to a weak ATP-site fragment could yield a selective bivalent CK2 inhibitor, since the αD pocket had not been observed in other kinases.

**Figure 6:**
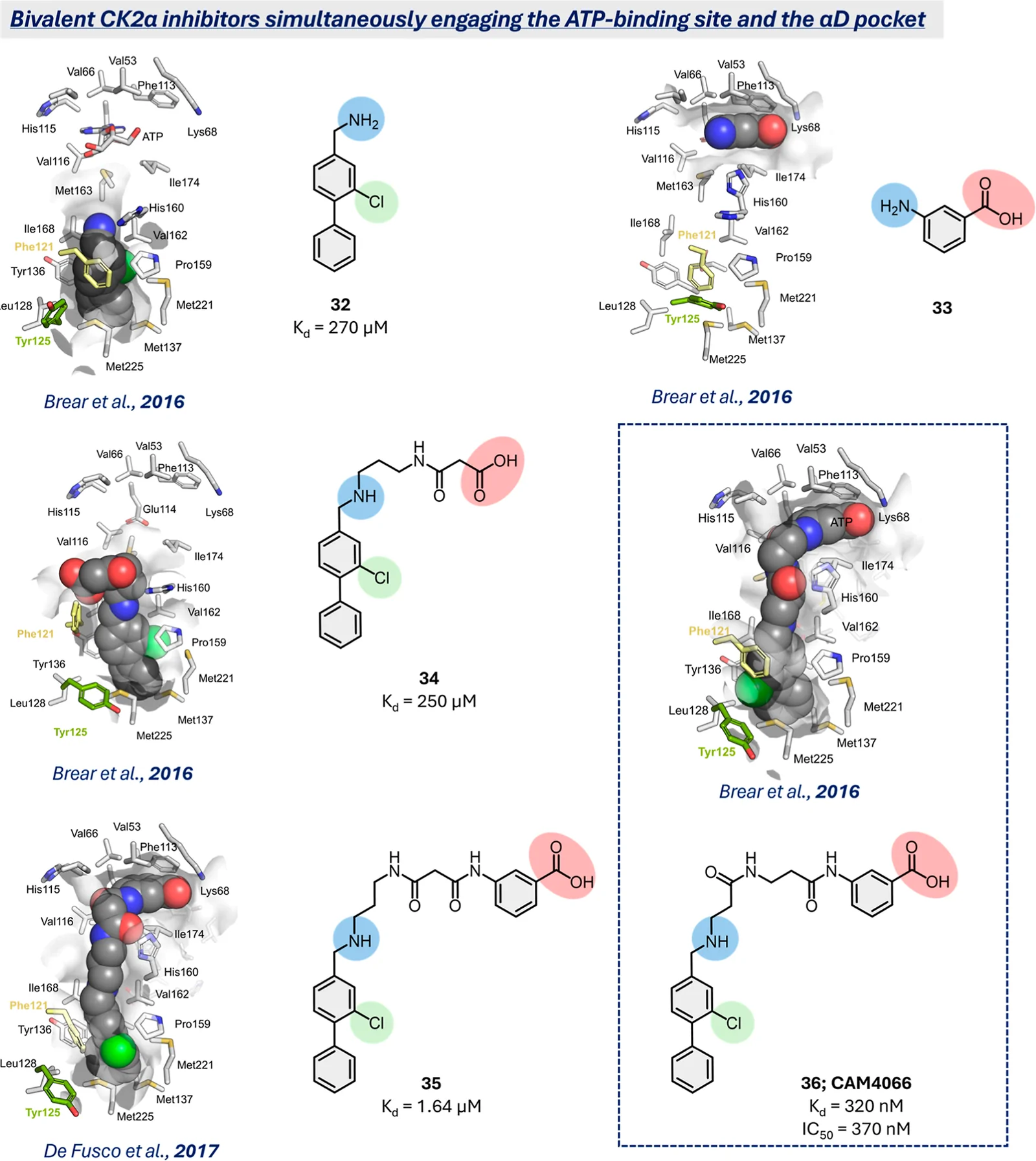
Development of the bivalent ligand CAM4066 (**36**), which engages both the CK2α ATP-binding site and the αD pocket and displays substantially improved potency. Fragment **32** occupied the deep hydrophobic αD pocket more effectively. Fragment **33** targeted the ATP-binding site. Fragment **34**, generated by linking **32** to a seven-atom linker, is shown in PDB: 5MO6. Compound **35**, created by joining fragments **33** and **34**, was the first design spanning both sites. CAM4066 (**36**) was the final proof-of-concept inhibitor utilizing the new cryptic pocket. In the crystal structure figures, side chains of all the residues in the ATP site and in the αD site are shown as sticks and labeled. Phe121 and Tyr125, that move significantly depending on the αD binder, are colored yellow and green, respectively. Color coding: oxygen as red, chlorine as green, nitrogen as blue.

From a crystallographic screen of a 384-fragment library, a weakly binding 3-aminobenzoic acid **33** was selected as the ATP-site moiety, interacting with catalytic Lys68 but not with the hinge region of CK2α (Figure 6). Initially, our rationale was that the affinity of a bivalent inhibitor should be lower for the ATP site than for the αD pocket, in order to ensure that the compound’s selectivity was dictated by engagement of the poorly conserved cryptic pocket.

Linker optimization was then undertaken to connect the αD and ATP-binding fragments without disrupting binding of either of the end moieties. A rigid or overly flexible linker would compromise dual occupancy either through entropic penalties or by introducing unwanted conformational strain. Structure-guided development of linkers from **32** toward the ATP site resulted in compound **34**, a derivative with a seven-atom linker, that bound CK2α with a similar affinity to **32** (Figure 6). The amide group in the linker formed a key hydrogen bond with the backbone carbonyl of His160. Analyses of the crystal structures showed good alignment of the terminal carboxylic acid of **34** with the amino group of **32,** which, when linked via an amide, resulted in **35**, which had modest affinity (K_d_ = 1.64 μM), attributed to geometric strain caused by tautomerisation at a carbonyl region of the linker (Figure 6). Relocating the second amide within the linker alleviated steric constraints, yielding CAM4066 (**36**, Figure 6), which bound CK2α with a K_d_ of 320 nM and inhibited its enzyme activity with an IC_50_ of 370 nM.

When profiled against 52 kinases at 2 μM (∼6 times above the IC_50_), **36** displayed exceptional selectivity, significantly inhibiting only CK2α, with a Gini coefficient of 0.82, the highest reported for a CK2 inhibitor at the time. However, its poor cell permeability, attributed to the terminal carboxylate group, prevented measurable effects on cell viability. To remove the charge, a methyl ester prodrug of **36** was synthesized, with the expectation that esterases in the cell hydrolyzed the prodrug to release the active **36**. Treatment of HCT116, Jurkat, and A459 cancer cells with this ester prodrug of **36** resulted in reduced viabilities of the cells, with GI_50_ values of 9, 6, and 20 μM, respectively. These values were comparable to those obtained with **30** (GI_50_ = 5, 5, and 17 μM, respectively). Although **30** remained the more potent inhibitor in enzymatic assays, the drop-off from biochemical IC_50_ to cellular GI_50_ was markedly smaller for **36**. Given that the larger potency drop-off observed for CX-4945 has been attributed in part to limited cellular permeability, the improved translation for **36** likely reflects more efficient cellular delivery of the ester prodrug and subsequent intracellular target engagement.

Overall, **36** represented a major advance by demonstrating that simultaneous engagement of the ATP site and the cryptic αD pocket by an orthosteric-cryptosteric bivalent inhibitor could provide potent and highly selective CK2α inhibition. This work provided a compelling proof-of-concept for targeting the αD pocket, laying the foundation for future strategies combining ATP-site occupation with engagement of unique, kinase-specific secondary sites. However, while **36** was a pivotal development in selectively targeting CK2, its reliance on a prodrug form for cellular activity and the susceptibility of its amide bonds to proteolytic cleavage limited its suitability as a drug. To overcome these drawbacks, we shifted focus to inhibiting CK2α through exclusive engagement of the αD site, reasoning that this approach could preserve selectivity since the site is unique to CK2.^56^

Starting from fragment **32** in the αD pocket, SAR studies examined substitutions on both aromatic rings and at the amine. Introducing an ethyl group at the 2-position of the lower ring gave compound **38** (K_d_ = 17 μM), while optimization of the upper ring produced the dichloro analogue **37**, guided by the observation of a dual conformation of **32** (K_d_ = 12 μM) in crystal structures (Figure 7A). Combining these features yielded compound **39**, which bound with a K_d_ of 7 μM, around 40-fold stronger than **32**, but did not displace ATP or inhibit CK2 activity (Figure 7A). The crystal structure of **39** in complex with CK2α shows how the ligand is interacting with the pocket. This work also demonstrated the malleability of the αD pocket, allowing for optimization of both binding and physicochemical properties.

**Figure 7:**
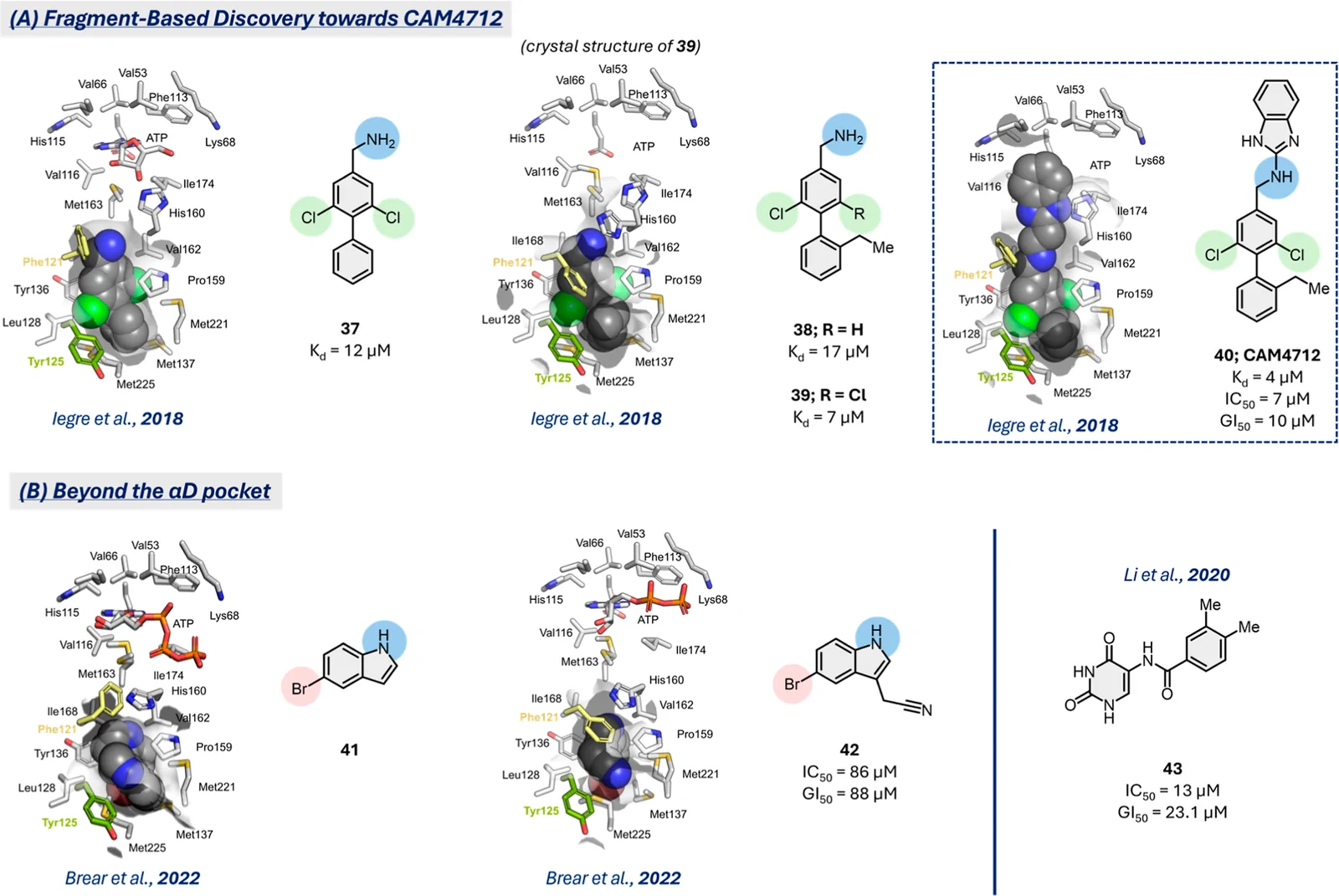
Other modalities of αD binding inhibitors. (A) Fragment-based development of CAM4712 (**40**), an αD-site inhibitor that blocks CK2 activity without engaging the ATP site. Compound **37**, incorporating a second *meta*-chloro substituent on the upper ring into **32**, bound more tightly than its parent (PDB: 5OTR). Compound **38**, bearing an ethyl group at the 2-position of the lower ring of **32**, showed improved αD-pocket binding (PDB: 5ORJ). Compound **39**, combining both modifications, further increased affinity (PDB: 5OTZ) but did not displace ATP and therefore failed to inhibit CK2. CAM4712 (**40**), produced by attaching a benzimidazole to the amine of **39**, bound with higher affinity; interactions with His160 and Met163 induced conformational changes that blocked ATP access. (B) Development of an αD-pocket fragment extending into pocket 2. Fragment **41** bound in the αD pocket without affecting ATP binding in two conformations (bound in two copies in the αD site, hence it looks like two compounds), revealing a second pocket (PDB: 7ZY2). Optimisation yielded fragment **42**, which reached into pocket 2 (PDB: 7ZY0). Compound **43**, a uracil-based αD-pocket inhibitor, was identified via virtual screening.

Incorporation of a benzimidazole at the amine afforded CAM4712 (**40**; Figure 7A), binding with a K_d_ of ∼4 μM and inhibiting CK2 with an IC_50_ of 7 μM (GI_50_ = 10 μM in HCT116 cells). The benzimidazole engaged in π–π stacking with His160 and interacted with Met163. It blocked the entrance to the ATP site and simultaneously induced a conformational change in the Met163 side chain that narrowed the ATP site. Although **40** showed lower potency and selectivity than **36**, it possessed improved physicochemical properties, requiring no prodrug modification and exhibiting greater rigidity and permeability. Collectively, **40** demonstrated that an αD-pocket inhibitor can achieve both biochemical and cellular inhibition of CK2 independently of direct ATP-site engagement.

Building on this work, we conducted further screening to identify improved αD-pocket inhibitors.^57^ Starting from fragment **41**, which bound exclusively within the αD pocket without affecting the ATP site, modifications of this were screened by crystallography and kinase assay. Fragment **42** emerged as the most promising hit, with an IC_50_ of 86 ± 24 μM (Figure 7B). The nitrile substituent of **42** extended into a hydrophobic “pocket 2” offering a new site for optimization. Although **42** did not displace ATP in co-crystal structures, it inhibited proliferation in HCT116, Jurkat, and A549 cells (GI_50_ of 88 ± 7, 55 ± 2, and 29 ± 7 μM, respectively), with consistent potency between enzymatic and cellular assays indicating good permeability. Target engagement was confirmed by reduced phosphorylation of AKT1 and CDC37. As the first αD-site inhibitor to act allosterically, **42** demonstrates that pocket engagement can indirectly impair catalysis even without disrupting ATP binding, providing a foundation for future design.

Inspired by these findings, Zhong and co-workers later employed structure-based virtual screening using the CK2α–compound complex **39** as a template.^58^ Using Alloscore to benchmark αD binders,^59^ they computationally filtered the ChemBridge library to 20 possible high-affinity candidates.^60^ Docking analyses narrowed these to six compounds predicted to engage Asn118, Pro159, or Val162. Biochemical testing identified compound **43** as the most effective (IC_50_ = 13.0 μM), suppressing A549 lung cancer cell proliferation (GI_50_ = 23.1 μM; Figure 7B). Although its potency was modest compared to **40** and lacked crystallographic confirmation of the binding mode, the study introduced a novel uracil-based scaffold that could potentially bind the αD pocket. This computational approach provided a complement to fragment-based discovery for future inhibitor optimization.

## 5 From Academic Discovery to Clinical Translation

Although substantial progress had been achieved with αD-pocket inhibitors, their potency remained lower than that of bivalent compound **36**. Consequently, efforts continued to develop dual-site inhibitors engaging both the αD pocket and the ATP-binding site. In one such study, the Krimm group reported^61^ AB668 (**44**), in which an indole moiety occupied the ATP site while a benzyl group bound the αD pocket (Figure 8).

**Figure 8:**
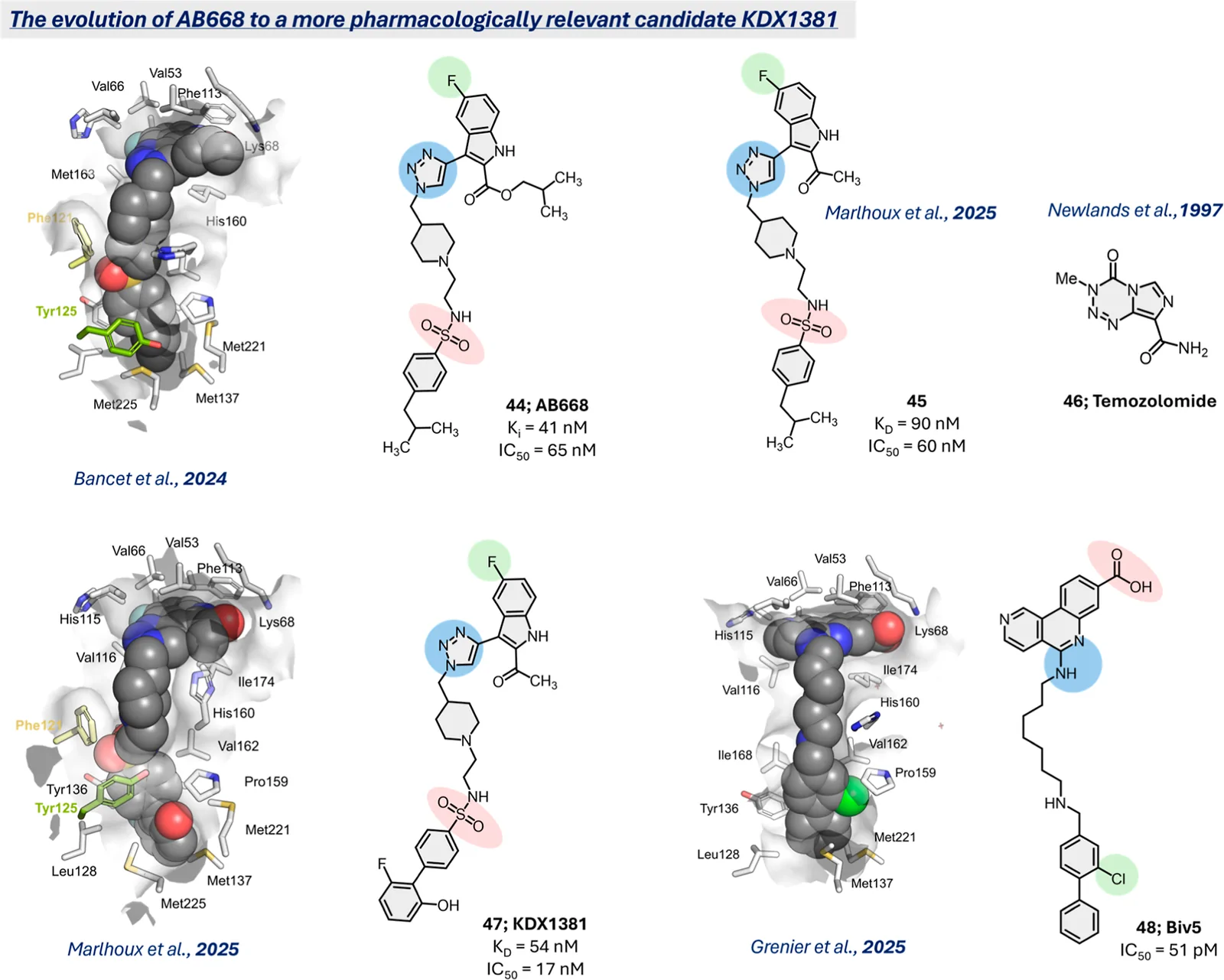
Development of bivalent CK2α inhibitors KDX1381 (**47**) and Biv5 (**48**). AB668 (**44**), a bivalent CK2 inhibitor simultaneously engaging the ATP-binding site and αD pocket (PDB: 8C5Q). Derivative **45**, in which the carboxylate group of AB668 was replaced by a methyl ketone, showed improved solubility. Temozolomide (**46**), a DNA-alkylating agent used in chemotherapy in combination with KDX1381 (**47**), a potent bivalent CK2 inhibitor exhibiting enhanced solubility and metabolic stability (PDB: 9HPH). Biv5 (**48**) combines an analogue of **30** as the ATP-site ligand with a 2-chloro-[1,1′-biphenyl] αD-pocket ligand, achieving subnanomolar CK2 inhibition and strong antiviral activity against SARS-CoV-2 and other β-coronaviruses.

**44** inhibited CK2 with a K_i_ of 41 nM and an IC_50_ of 65 nM, representing improved potency relative to **36**. When screened against 468 kinases at 2 μM concentration through an active site-directed competition binding assay, **44** showed remarkable selectivity for CK2α, with only RPS6KA5 inhibited by more than 50%. In cancer cell lines, **44** induced apoptosis broadly and was particularly effective in 786-O and A375 cells, outperforming **30** and SGC**-**CK2-1, another ATP-site inhibitor.^62^ CK2 inhibition persisted after compound removal, unlike with **30**, suggesting durable target engagement. Consistent with this, **44** reduced phosphorylation of CK2 substrates and downregulated genes linked to proliferation, confirming it as a potent, pharmacologically improved successor to **36**. However, despite its potency, **44** suffered from poor solubility and metabolic stability. Optimisation of the ATP-site indole revealed the C2 carbonyl and 5-fluoro substituents as essential; replacing the C2 isobutyl ester with a methyl ketone improved stability while retaining potency, yielding analogue **45** (Figure 8).^63^ Refinement of the αD-pocket binder through biphenyl substitution produced KDX1381 (**47**), containing *ortho*-fluoro and *ortho*-hydroxy groups. **47** maintained the potency of **44** and **45** but displayed markedly improved solubility and hepatocyte stability. In 786-O cells, **47** reduced CK2 substrate phosphorylation, induced apoptosis in 786-O and HCT116 lines, and suppressed colony formation in HCT116 cells. In xenografts, it achieved ∼90% tumor growth inhibition (TGI) with good tolerability. Combination with vascular endothelial growth factor receptor tyrosine kinase inhibitors (VEGFR-TKIs) or Temozolomide (**46**) enhanced pathway suppression and efficacy in glioma models. Overall, **47** matched the potency of **44** but offered superior pharmacokinetics. Up to this point, bivalent inhibitors employed either a benzoic acid in **36** or indole in **44**, **45** and **47** to engage the ATP site. Seeking greater potency, the Krimm group used a scaffold based on **30** (Figure 5B), known for high CK2α affinity,^64^ and evaluated antiviral potential. Optimisation of linker and αD-pocket moieties produced Biv5 (**48**), combining the **30**-derived ATP ligand with the 2-chloro-[1,1′-biphenyl] αD-pocket inhibitor developed by our groups (Figure 8).^19,20^ **48** achieved subnanomolar CK2 inhibition and suppressed SARS-CoV-2 replication (IC_50_ = 51 ± 10 pM), though it lacked full selectivity, also targeting DRAK2.

To address remaining gaps in potency, selectivity, and pharmacokinetics, the Hyvönen and Spring groups collaborated with Apollo Therapeutics to develop a clinical candidate.^65–71^ We noted that while αD-pocket engagement should drive selectivity, early iterations like **36** failed to reach the required affinity.^19,20^ Nevertheless, dual-site inhibitors also present broader optimization challenges, as their increased molecular size and structural complexity can adversely affect permeability, solubility, metabolic stability, and pharmacokinetic behavior. Their development therefore requires careful optimization beyond potency and biochemical selectivity alone. The project team hypothesized that replacing the benzoic acid moiety of **36**, which occupied the ATP-binding site, with a stronger ATP-site ligand could yield a more potent inhibitor, in which affinity would be driven by deeper engagement of the ATP-binding site while selectivity would still arise from occupancy of the unique αD pocket.

To identify an appropriate ATP-binding fragment, all kinase ligands available in the Protein Data Bank were overlaid onto the crystal structure of **36** bound to CK2α to identify ligands with vectors compatible for optimal interactions in the ATP-binding site. This computational screen led to the design of approximately two hundred compounds, which were subsequently narrowed down to 28 representative molecules tested for both potency and selectivity, aiming for higher potency than **36** and higher selectivity than **30**. This screening effort led to the discovery of three chemotypes: one containing a **30**-like tricyclic scaffold (**49**), one featuring a pyrazole motif (**50**), and one incorporating an indazole motif (**51**; Figure 9).

**Figure 9:**
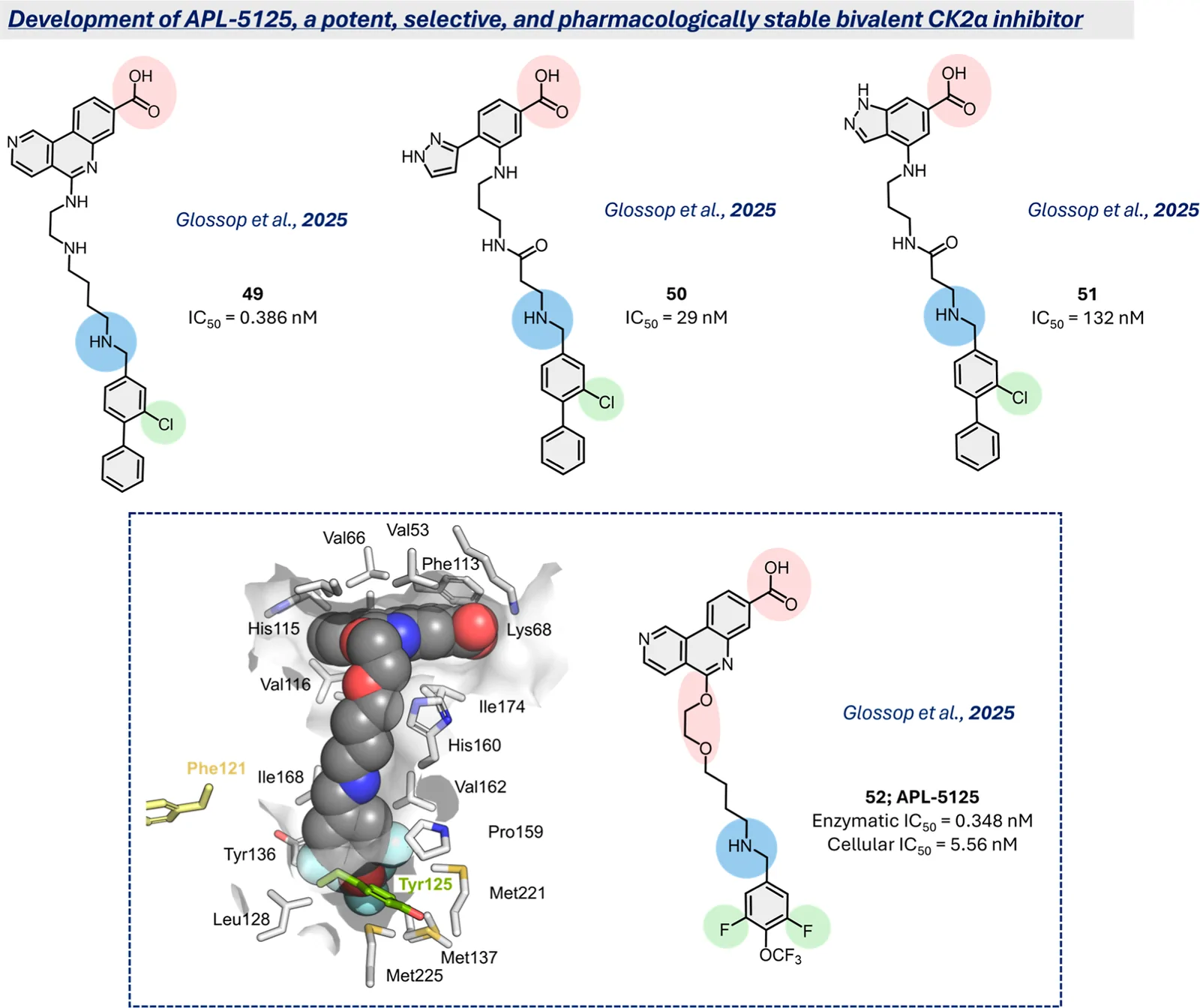
Development of APL-5125 (**52**), a potent, selective, and metabolically stable bivalent CK2α inhibitor. Compound **49** emerged as the most active analogue from the initial tricyclic ATP-site series fused to the biaryl αD ligand. Compound **50** was the initial analogue from the pyrazole-based series, and compound **51** from the indazole-based series, each linked to the biaryl αD ligand. APL-5125 (**52**), the lead molecule from SAR optimization, contains a tricyclic ATP-site ligand, a 3,5-difluoro-4-OCF_3_ αD-pocket group, and a linker with a central ether (PDB: 7I8K). It showed excellent translation from in vitro potency to in vivo efficacy and is now in clinical trials.

The latter two represented novel hinge-binding motifs for CK2α inhibition, broadening the structural scope for targeting this kinase. The tricyclic series exhibited the highest potency, with the motif extending deeper into the ATP-binding site than the benzoic acid of **36**, forming additional interactions with Val116 and Glu114. Moreover, the 90° turn of the linker at the hinge region enabled the benzylic amine of the αD-pocket ligand to form hydrogen bonds with the backbone carbonyls of Val162 and Pro159, thereby anchoring the molecule effectively. Consequently, further SAR optimization was pursued using the tricyclic scaffold as the ATP-binding site ligand. Initially, this work focused on isosteres of the carboxylic acid (to modulate physicochemical properties) and modifications to the linker (to reduce basic centers and/or rotatable bonds). Replacement of the carboxylate was successfully achieved with a primary amide and a tetrazole, which were both very potent in the biochemical assay, but the tetrazole had weak potency in cellular assays, presumably due to poor permeability. Focus on the linker revealed that a seven-atom distance between the benzylic amines was optimal, and that up to two amino groups could be replaced, with an ether linkage outperforming amide or other alternatives. The benzylic NH adjacent to the αD fragment was found to be essential for activity. Subsequent optimization focused on improving the pharmacokinetic profile. To enhance metabolic stability, the lower ring of the biaryl motif was removed, and substituents were introduced to the remaining phenyl ring to reduce lipophilicity and/or block metabolism. These efforts culminated in the identification of the 3,5-difluoro-4-OCF_3_ analogue, which provided the best balance between potency, selectivity, and metabolic stability. Combining this αD-pocket ligand with the seven-atom ether-linked spacer and the tricyclic ATP-binding site ligand resulted in the discovery of APL-5125 (**52**; Figure 9).

Compound **52** exhibited a CK2α IC_50_ of 0.348 nM in enzymatic assays and displayed a high degree of selectivity—only five of 403 kinases tested were inhibited by more than 65% at 100 nM and follow-up profiling against these hits confirmed the high selectivity of **52**. In cellular assays using the HCT116 line, **52** was over 150-fold more potent than **30** in suppressing phosphorylation of the CK2 substrate p-AKT (S129). These findings indicate that biochemical kinase selectivity alone may not fully predict cellular pharmacology, as differences in binding mode and physicochemical properties can influence cellular exposure, target engagement, pathway modulation, and ultimately therapeutic activity. Furthermore, **52** demonstrated high metabolic stability in human liver microsomes and hepatocytes, and acceptable permeability in Caco-2 cells.

Encouraged by its in vitro profile, **52** was advanced to in vivo studies. In rats, oral absorption was initially modest (16% and 36% in males and females, respectively), but coformulation with Kolliphor^72^ markedly improved oral bioavailability to 66% and oral absorption to 78% in male rats. A similar improvement was observed in dogs. In mice bearing HCT116 xenografts, oral administration of **52** led to significant tumor growth regression and an impressive in vivo IC_50_ of 2.9 nM, demonstrating excellent translation from enzymatic to cellular and animal models. The data obtained on compound **52** from different assays are summarized in Table 1. These results collectively validated the bivalent design strategy as a viable route to drug-like CK2 inhibition and have propelled **52** into Phase 1/2 clinical trials for patients with advanced solid tumors (ClinicalTrials.gov identifier: NCT06399757).^73^ APL-5125 (**52**) is therefore the third CK2-targeting agent to enter clinical evaluation, following CX-4945 and CIGB-300,^5,74–76^ which acts by binding to and inhibiting the phosphorylation of the phosphoacceptor domains of certain CK2 substrates.

**Table 1:**
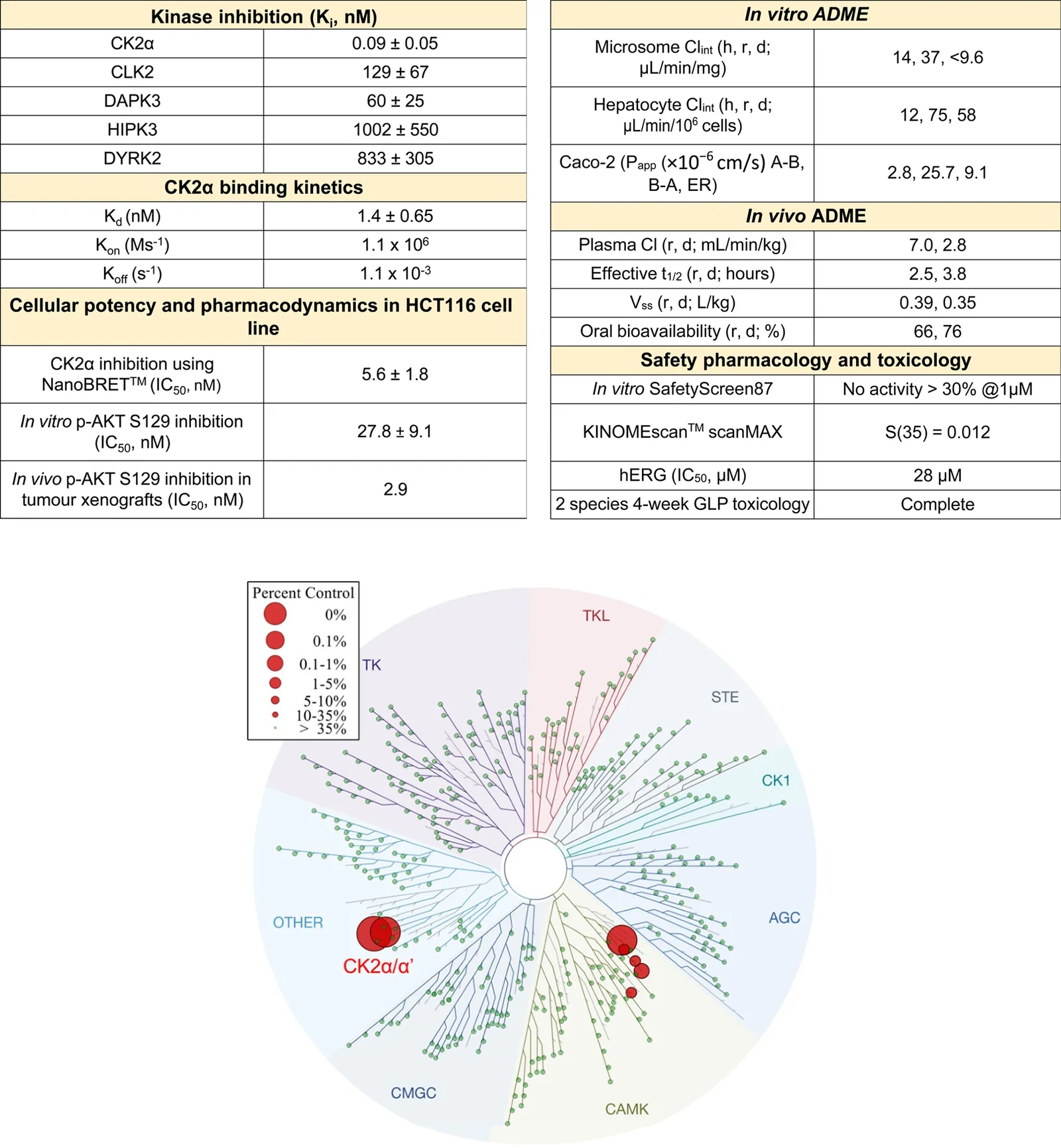
Summary of the Potency, Pharmacodynamic, Pharmacokinetic, and Safety Data Obtained from the Studies on Compound **52**, and KINOMEscan scanMAX Profiling TREEspot Analysis of the Activity of Compound **52** against a Range of Kinases, Demonstrating its High Selectivity for CK2^a^

a TREEspot analysis reproduced from ref 71. Available under a CC-BY 4.0 license. Copyright 2025 Glossop et al., American Chemical Society.

## 6 Outlook and Perspectives

Progress toward selective CK2 inhibition has been driven by successive, interconnected advances across multiple laboratories, with our groups and collaborators contributing pivotal discoveries that reshaped the field. Early studies by others first demonstrated that the CK2 holoenzyme could be perturbed through interface-directed small molecules and cyclic peptides, laying essential groundwork. Building on this foundation, we validated the CK2α/β interface as a druggable target through fragment-derived binders such as CAM187 (**9**), providing the first crystallographic evidence for small-molecule engagement of this site. Complementary efforts on conformationally constrained peptides CAM7117 (**17**) and P8C9 (**19**) further demonstrated that interface disruption could be achieved with stable, cell-permeable scaffolds.

Subsequent validation of the cryptic αD pocket as a ligandable site marked a turning point, revealing a structural feature that could be exploited for kinase selectivity. This advance enabled the development of dual-site ligands such as CAM4066 (**36**) and αD-exclusive inhibitors such as CAM4712 (**40**), while independent follow-up studies further expanded this design concept. Subsequent studies by other groups, including those describing AB668 (**44)**, KDX1381 (**47**), and Biv5 (**48**), further validated and refined this approach, producing pharmacologically advanced bivalent inhibitors and demonstrating the translatability of αD-pocket engagement. This trajectory culminated in APL-5125 (**52**), developed in collaboration with Apollo Therapeutics: an orthosteric-cryptosteric (dual-site) inhibitor combining subnanomolar potency, exceptional selectivity, and in vivo efficacy that has progressed into clinical trials. This achievement exemplifies how structure-guided, collaborative academic research can evolve into therapeutically relevant outcomes. Looking ahead, future opportunities lie in integrating αD-pocket-directed ligands with emerging modalities such as PROTACs, antibody–drug conjugates, and covalent inhibitors, as well as in probing CK2’s broader signaling context through degraders and chemical probes. The CK2 story, spanning interface disruption, cryptic-pocket validation and exploitation, and clinical translation, demonstrates how iterative and cross-disciplinary design can convert a challenging kinase into a validated therapeutic target, while underscoring the value of collaboration and continuity across generations of research.

## Abbreviations

ADP: adenosine diphosphate
AM: acetoxymethyl
ATP: adenosine triphosphate
CK2: casein kinase 2
CPP: cell-penetrating peptide
CuAAC: copper-catalyzed azide–alkyne cycloaddition
FBDD: fragment-based drug design
GI_50_: growth inhibition 50%
HEK: human embryonic kidney
HRT18: human rectal tumor cell line
IC_50_: half-maximal inhibitory concentration
K_d_: equilibrium dissociation constant
K_i_: inhibition constant
PDB: Protein Data Bank
PEG: polyethylene glycol
PROTACs: proteolysis-targeting chimeras
SAR: structure–activity relationship
TAT: *trans*-activating transcriptional activator
TGI: tumor growth inhibition
VEGFR-TKI: vascular endothelial growth factor receptor tyrosine kinase inhibitor.
